# The Efficacy of Long-Term Chinese Herbal Medicine Use on Lung Cancer Survival Time: A Retrospective Two-Center Cohort Study with Propensity Score Matching

**DOI:** 10.1155/2021/5522934

**Published:** 2021-08-23

**Authors:** Li-Li Xu, Su-Fang Zhang, Yu-Li Wang, Ying-Bin Luo, Zhi-Hong Fang, Yuan Fang, Rong-Zhong Xu, Peng Guo, Jian-Chun Wu, Yan Li

**Affiliations:** ^1^Department of Oncology, Shanghai Municipal Hospital of Traditional Chinese Medicine, Shanghai University of Traditional Chinese Medicine, Shanghai, China; ^2^Department of Traditional Chinese and Western Medicine, Shanghai Pulmonary Hospital, Tongji University School of Medicine, Shanghai, China

## Abstract

**Objective:**

To explore the efficacy of long-term use of Chinese herbal medicine (CHM) on survival time of lung cancer.

**Methods:**

We conducted a retrospective cohort study on lung cancer patients. A propensity score matching (PSM) was performed to balance the covariates. Progression-free survival (PFS) was the primary endpoint and overall survival (OS) was the secondary endpoint. Patients who received CHM therapy from the initial date of diagnosis of lung cancer were included in the CHM group. Patients who were not treated with CHM during the same interval were categorized in the control group. A Cox regression model was used to explore the prognostic factors related to lung cancer. Hazard ratios of different subgroups were also analyzed.

**Results:**

A total of 1134 patients were included in our study: 761 patients were in the CHM group and 373 patients were in the control group. After PSM, the mPFS and mOS in the CHM group were 70.4 months and 129.1 months, respectively, while the mPFS and mOS in the control group were 23.8 months and 99.7 months, respectively. The results of survival analysis on each stage demonstrated that patients may benefit from the long-term CHM treatment especially for patients with early stage. One-year to ten-year progression-free survival rates in the CHM group were higher than those in the control group (*p* < 0.001). COX multivariate regression analysis indicated that CHM treatment, female, low age at diagnosis, early tumor stage, and surgery were independent protective factors against recurrence and metastasis of lung cancer. Subgroup analysis showed that CHM treatment could reduce the risk of recurrence and metastasis in each subgroup (*p* < 0.01).

**Conclusion:**

Long-term CHM treatment with the *Fuzheng Quxie Formula,* which can be flexibly applied in the course of lung cancer treatment, not only has a positive influence on the progression-free survival time of lung cancer patients, but also reduces the risk of recurrence and metastasis of lung cancer.

## 1. Introduction

Lung cancer is a public health concern worldwide and remains the highest incidence rate of cancer, with 228,820 new cases and 135,720 deaths predicted in 2020 according to statistical results published by GLOBOCAN 2020 [1]. With the population aging and rapid socioeconomic development, China is now facing a huge health, financial, and societal challenge in cancer prevention and treatment. Statistics from the National Central Cancer Registry estimated that the total number of newly diagnosed cases of lung cancer in 2015 was about 787,000. Meanwhile, lung cancer accounts for 20% of all cancer cases [2]. Surgical interventions for cancer have evolved rapidly in the last two decades. For example, the percentage of video-assisted thoracic surgery (VATS) among thoracic surgeries rose over 30% from 2008 to 2014 in the National Cancer Center and up to 80% between 2014 and 2019 [3]. Although surgery, radiotherapy, and systemic therapy (including chemotherapy, targeted therapy, and immunotherapy) have all been developed for the treatment of cancer, the overall cure rate and survival rate of lung cancer remain unsatisfactory. For example, cisplatin can induce the apoptosis of platelets and can also lead to impaired platelet function by upregulating Bax, Bak, and Bcl-2 and downregulating Bcl-xl [4]. Gemcitabine can increase the apoptosis-promoting protein content and accelerate the process of platelet apoptosis by reducing the activity of Bcl-xl in patients [5]. Although targeted drugs are generally safe, there are also reports in the literature that they can cause thrombocytopenia, rashes, diarrhea, etc. [6–8]. Postoperative adjuvant chemotherapy is an effective way to reduce the recurrence rate. There are differences in the efficacy of adjuvant chemotherapy between different patients. The long-term survival rate after surgery is low. The 5-year survival rate after lung cancer surgery is 22.0%–47.3% [9]. Actually, prolonging the long-term survival is the ultimate goal of all treatments for patients with advanced-stage disease.

Therefore, developing more effective therapeutic strategies for lung cancer remains an important challenge. Currently, Traditional Chinese Medicine (TCM) has become an adjuvant treatment method in the comprehensive treatment of lung cancer [10]. Several studies have reported that Chinese herbal medicine (CHM) treatment could strengthen *zheng-qi* during the postoperative period, reduce adverse effects of chemotherapy and radiotherapy, and prevent relapse and metastasis [11–13]. The TCM theory provides a macroscopic view of biological phenomena. Chinese herbal formulae are prescribed under the guidance of syndrome differentiation which includes comprehensive analysis after observation, auscultation, olfaction, interrogation, and pulse-feeling and palpation.

The *Fuzheng Quxie Formula* is a Chinese herbal prescription invented by Professor *Li* for the treatment of respiratory system tumors, which underpinned Chinese National Medical Professor *Jiaxiang Liu*'s core principle ‘strengthening vital *qi* to treat cancer' [14]. Clinical trials found that the *Fuzheng Quxie Formula* could improve progression-free survival (PFS) time and immune function of patients with lung cancer [15, 16]. Additionally, experimental research suggested that the *Fuzheng Quxie Formula* likely inhibited the migration and invasion of human adenocarcinoma A549 cells via regulation of TGF-*β*3mRNA expression [17]. Efficacy of the *Fuzheng Quxie Formula* in improving the outcomes of lung cancer patients appears to be demonstrated. However, neither large-sample research nor long-term clinical observation has been conducted, and survival time associated with combined treatment with the *Fuzheng Quxie Formula* and conventional medicine in lung cancer patients has not been evaluated.

Thus, the current longitudinal cohort study aimed to determine whether the addition of CHM to conventional medicine prolongs overall survival (OS) and PFS compared with conventional medicine in patients with lung cancer.

## 2. Material and Methods

### 2.1. Study Design and Participants

This two-center, retrospective cohort study collected clinical data from lung cancer patients who received treatments from the Shanghai Pulmonary Hospital and Shanghai Municipal Hospital of Traditional Chinese Medicine between January 1, 2005, and December 30, 2020. This study was approved by the Ethical Committee of Shanghai Municipal Hospital of Traditional Chinese Medicine (2019SHL-KY-44). The treatment protocols were carried out in accordance with the principles of the Helsinki Declaration.

### 2.2. Case Selection Criteria

The inclusion criteria were as follows: (1) having a diagnosis of primary lung cancer, confirmed histologically or cytologically; (2) age ≥ 18 years; (3) agreement to participate in the study, which included regular follow-up; (4) Eastern Cooperative Oncology Group (ECOG) performance ≤ 2 [18].

The exclusion criteria were as follows: (1) diagnosis of secondary lung cancer; (2) presence of severe/uncontrolled systemic diseases including gastrointestinal dysfunction, bleeding, cardiac dysfunction, endocrine dysfunction, or infection; (3) having other malignant tumors; (4) pregnancy; (5) severe fragmentation of follow-up data; (6) Karnofsky (KPS) score ＜ 60.

### 2.3. CHM Procedure

Patients in the control group from Shanghai Pulmonary Hospital received surgery, radiotherapy, chemotherapy, targeted therapy, and optimal supportive therapy according to the National Comprehensive Cancer Network (NCCN).

Patients in the CHM group from Shanghai Municipal Hospital of Traditional Chinese Medicine took modified *Fuzheng Quxie Formula* for at least six months, with the specific drug composition as follows: Radix Astragali (*Shenghuangqi*, 30 g), Rhizoma Atractylodis Macrocephalae (*Shengbaizhu*, 9 g), Poria (*Baifuling*, 15 g), Rhizoma Dioscoreae (*Huaishanyao*, 18 g), Semen Coicis (*Yiyiren*, 18 g), Pericarpium Citri Reticulatae (*Chenpi*, 9 g), Fructus Lycii (*Gouqizi*, 18 g), Fructus Ligustri Lucidi (*Nvzhenzi*, 18 g), Radix Glehniae (*Beishashen*, 15 g), Radix Ophiopogonis (*Maidong*, 15 g), Herba Hedyotis Diffusae (*Sheshecao*, 15 g), Herba Salviae Chinensis (*Shijianchuan*, 15 g), and Selaginella Doederleinii (*Shishangbai*, 15 g). The dosage and composition of the herbal were also adjusted by clinicians depending on the patient's clinical condition. For instance, patients with insomnia were treated with the addition of Semen Ziziphi Spinosae (30 g) and Concha Margaritifera (30 g). For patients with constipation, Fructus Cannabis (30 g) and Semen Pruni (30 g) were added. Patients who suffered from cancer pain were treated with the addition of Rhizoma Corydalis (15 g) and Radix Cynanchi Paniculati (30 g). For diarrhea, Semen Lablab Album (30 g) and Semen Nelumbinis (30 g) were added. For poor appetite and nausea, Fructus Crataegi (15 g), Roasted Fructus Hordei Germinatus (30 g), Caulis Bambusae in Taenia (9 g), and Flos Inulae (9 g) were added. Retinervus Citri Reticulatae Fructus (9 g) and Rhizoma Belamcandae (9 g) were added if patients felt dry and itchy throat. For patients with high blood pressure, Rhizoma Gastrodiae (15 g) and Ramulus Uncariae Cumuncis (15 g) were added. Herba Sedi (30 g) and Herba Hyperici Japonici (15) were used additionally for liver dysfunction. One experienced TCM physician, Professor *Li,* was assigned to syndrome differentiation.

CHM treatments were administered daily during the intervention period. For decoction, the herbs were soaked in water for thirty minutes, followed by boiling 450 ml of the solution. 150 mL of one set of herbs was administered three times per day, thirty minutes after each meal. Clinical research pharmacists took part in and supervised the procedures. The herbs were all provided by Chinese medicine pharmacies of Shanghai Municipal Hospital of Traditional Chinese Medicine. All herbal medicine was sourced from the same production area.

### 2.4. Follow-Up and Data Outcomes

All patients were followed from the date of diagnosis to the date of death or up to December 30, 2020. The date of death was determined from a database maintained by the Shanghai Municipal Center for Disease Control and Prevention of Cancer Patient Registration System. Additionally, telephone and clinical in-person visits were performed every six months.

The following data were collected: (1) basic information: name, age, gender, identification number, and contact information; (2) disease-related information: diagnosis date, first visit date, pathological stage, pathological category, location of tumor, operative treatment, type of treatment, including chemotherapy, radiotherapy, interventional therapy, targeted therapy, and TCM therapy; (3) personal history: smoking and drinking history; (4) family history: tumor-related family history.

The primary endpoint was PFS, which was the time from the start of treatment to metastasis recurrence or death. The secondary endpoint was OS, which was defined as the time from the beginning of treatment to the time of death.

### 2.5. Propensity Score Matching

To reduce intergroup selection bias between the CHM group and the control group, propensity score matching (PSM) was used [19]. A logistic regression analysis was performed to generate propensity scores, which included potential confounding variables. A multivariate Cox model was used to investigate confounding variables predicting median PFS and OS (mPFS/mOS). All patients were matched via a 1 : 1 protocol with a caliper width equal to 0.05 of the standard deviation of the logit of the propensity score and without any replacement.

### 2.6. Statistical Analysis

Stata software (version15.0, Stata Cooperation, College Station, TX, USA) was used for data analysis. Continuous variables were summarized using means and standard deviations, and categorical variables were summarized using counts and percentages. Differences between the baseline characteristics of CHM users and nonusers were analyzed with *t*-tests for continuous variables, and Pearson's *χ*^2^ test or Fisher's exact test for categorical variables. The cumulative survival probability for CHM users and nonusers was estimated using a Kaplan–Meier estimator with a log-rank test used to compare survival curves between groups. The cumulative recurrence of metastasis, annual metastasis rates from 1 to 10 years, mPFS, and mOS time were calculated for the two groups. A Cox proportional hazard regression model was used to assess the effect of independent factors on the survival prognosis of all patients. Statistical significance threshold was set at *P* < 0.05.

## 3. Results

### 3.1. Baseline Characteristics and Clinical Manifestations

**Figure 1:**
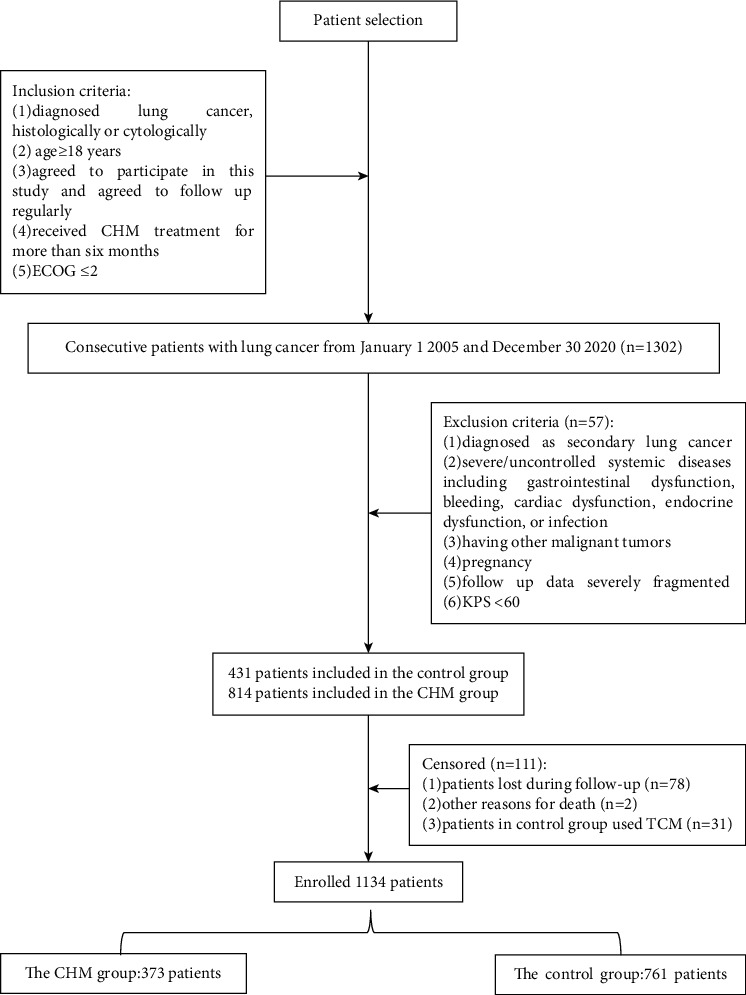
Flowchart of patient selection, inclusion, and exclusion.

Data from all 1302 patients with lung cancer were screened in our retrospective cohort study. Among these patients, 57 were excluded according to the exclusion criteria. An additional 111 patients were removed, including 78 patients who were lost during the follow-up period, 2 patients who died from diseases unrelated to lung cancer, and 31 patients in the control group who used TCM treatment after the study. Ultimately, 1134 patients were eligible for further analysis. The patient enrollment flowchart is shown in Figure 1. Baseline demographics and clinical characteristic of patients before and after PSM are presented in Table 1. Before PSM, there were 373 patients in the control group and 761 patients in the CHM group. Several clinical variables exhibited significant between-group differences: gender, age, pathology, stage, surgery, chemotherapy, radiotherapy, interventional therapy, targeted therapy, family history, smoking, and drinking (*P* < 0.05). After PSM, there were 208 patients in control group and 208 patients in CHM group.

### 3.2. Between-Group Survival Analysis

**Figure 2:**
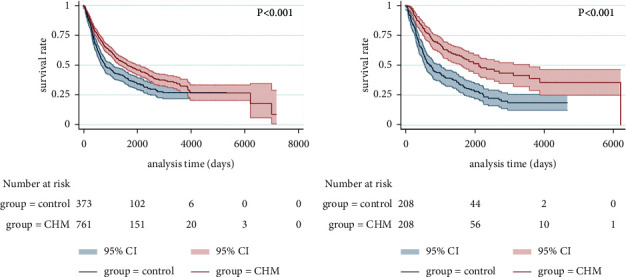
Kaplan–Meier curve of PFS of lung cancer patients before (a) and after (b) PSM.

**Figure 3:**
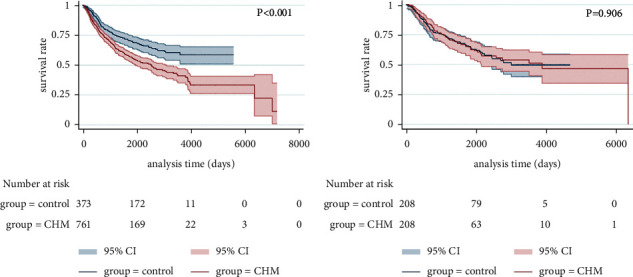
Kaplan–Meier curve of OS of lung cancer patients before (a) and after (b) PSM.

Before PSM, the median progression-free survival (mPFS) time of the control group and the CHM group was 26.6 months and 54.8 months, respectively (HR = 0.721, 95%CI:0.614–0.846, log-rank *p* < 0.001, Figure 2(a)). After PSM, the mPFS time of the control group and the CHM group was 23.8 months and 70.4 months, respectively (HR = 0.500, 95%CI:0.387–0.646, log-rank *p* < 0.001, Figure 2(b)). Before PSM, the median overall survival (mOS) time of the control group was not given. And the mOS time of the CHM group was 84.6 months (HR = 1.633, 95%CI:1.320–2.021, log-rank *p* < 0.001, Figure 3(a)). After PSM, the mOS time of the control group and the CHM group was 99.7 months and 129.1 months, respectively (HR = 0.981, 95% CI: 0.714–1.348, log-rank *p*=0.906, Figure 3(b)).

From the above results, we found that, after PSM, the CHM group showed a greater advantage over the control group in improving the PFS time of lung cancer.

### 3.3. Survival Analysis in Different Disease Stages

According to the above survival analysis and previous clinical experience, the survival time of lung cancer patients is quite different according to disease stages. Therefore, we further explored the efficacy of CHM treatment on both PFS and OS at each stage.

**Figure 4:**
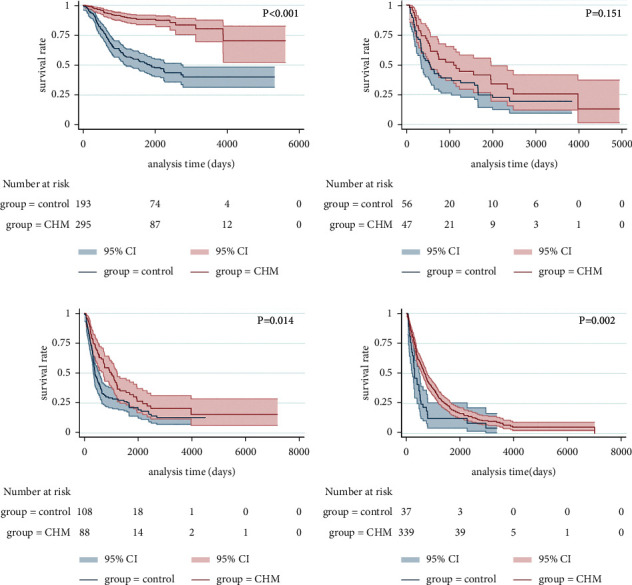
Kaplan–Meier curve of PFS for patients with stages I (a), II (b), III (c), and IV (d) (before PSM).

The mPFS time of stage I of the control group was 70.0 months and was not given in the CHM group, but there was still statistically significant difference between the two groups (*p* < 0.001, Figure 4(a)). The stage II mPFS time was 17.7 months in the control group and 36.4 months in the CHM group, respectively (*p*=0.151, Figure 4(b)). The stage III mPFS time was 13.3 months in the control group and 33.1 months in the CHM group, respectively (*p*=0.014, Figure 4(c)). The stage IV mPFS time was 10.0 months in the control group and 22.5 months in the CHM group (*p*=0.002, Figure 4(d)).

**Figure 5:**
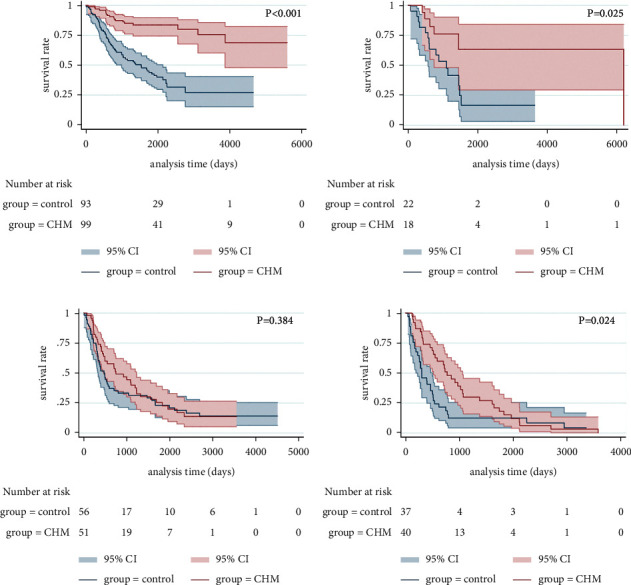
Kaplan–Meier curve of PFS for patients with stages I (a), II (b), III (c), and IV (d) (after PSM).

After PSM, the mPFS time of stage I of the control group was 51.1 months and was not given in the CHM group, but there was still statistically significant difference between the two groups (*p* < 0.001, Figure 5(a)). The stage II mPFS time was 36.6 months in the control group and 206.5 months in the CHM group, respectively (*p*=0.025, Figure 5(b)). The stage III mPFS time was 15.8 months in the control group and 24.8 months in the CHM group, respectively (*p*=0.384, Figure 5(c)). The stage IV mPFS time was 10.0 months in the control group and 25.7 months in the CHM group, respectively (*p*=0.024, Figure 5(d)).

**Figure 6:**
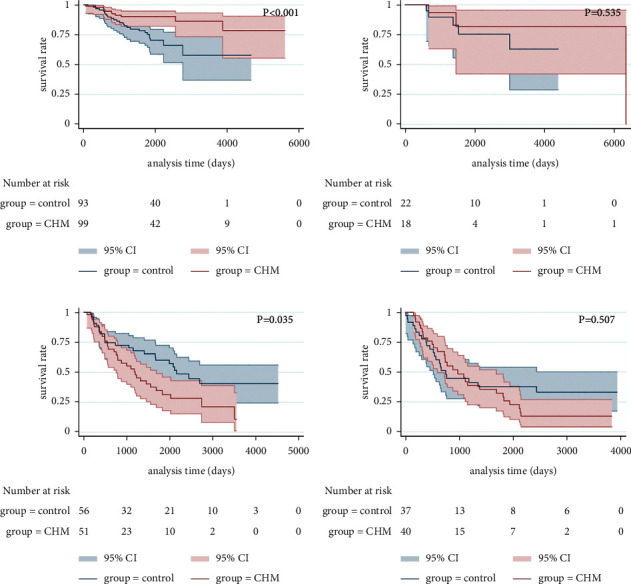
Kaplan–Meier curve of OS for patients with stages I (a), II (b), III (c), and IV (d) (after PSM).

After PSM, there was significant difference in the survival curve of stage I lung cancer (*p* < 0.001, Figure 6(a)), but there were no significant advantages in the survival curves of stage II (*p*=0.535, Figure 6(b)), stage III (*p*=0.035, Figure 6(c)), and stage IV lung cancer (*p*=0.507, Figure 6(d)).

### 3.4. Survival Rate

**Figure 7:**
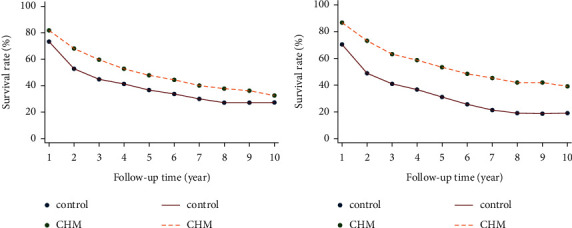
Comparison of progression-free survival rate between two groups before (a) and after (b) PSM.

The progression-free rates of the two groups during follow-up time were also calculated. As shown in Table 2, the 1∼10-year progression-free rate was significantly higher in the CHM group compared to the control group, both before (Figure 7(a)) and after PSM (Figure 7(b)).

### 3.5. Cox Regression and Subgroup Analysis of Hazard Ratio (HR)

A total of 13 factors were incorporated into the Cox regression with the outcome of recurrence and metastasis. Results of univariate analysis indicated that younger female patients taking CHM treatment with squamous cell cancer, earlier tumor stage, surgery, chemotherapy, radiotherapy, targeted therapy, family history, non-smoking history, and non-drinking history had longer PFS. As shown in Table 3, it showed that CHM treatment, female, low age at diagnosis, early tumor stage, and surgery were independent protective factors against recurrence and metastasis of lung cancer. Besides, HR of CHM for recurrence and metastasis was 0.721 (*P* < 0.001, 95%CI:0.614–0.846), which indicated that taking CHM treatment could significantly prevent the recurrence and metastasis of lung cancer.

**Figure 8:**
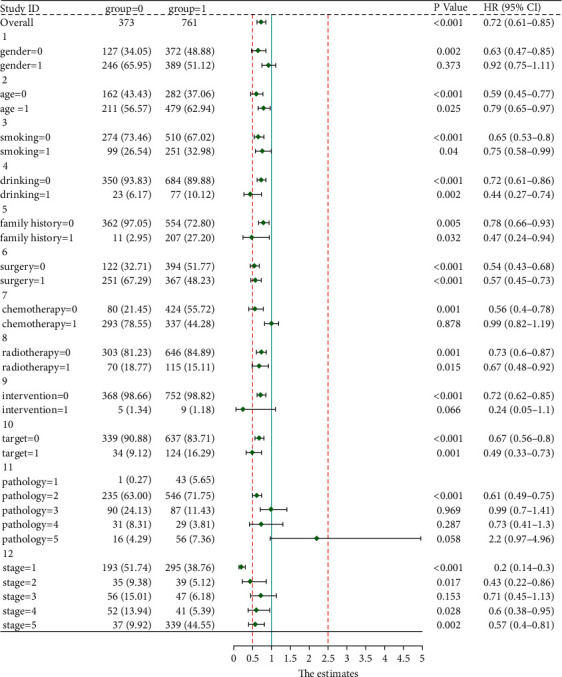
Forest plot of HR and 95% confidence interval between CHM and control group.

The subgroup analysis revealed that HRs for the entire CHM group were lower than that of the control group (HR = 0.72, 95%CI:0.61–0.85). Additionally, the HR of female patients younger than 60 years old with no smoking history, family history, no surgery, no chemotherapy, radiotherapy, targeted therapy, and stage I in the CHM group was significantly lower than that of the control group (Figure 8**).**

## 4. Discussion

CHM and conventional medicine were usually prescribed independently several years ago, and even more so 2 decades ago [20]. However, with the rapid growth of CHM-based evidence, integrating CHM into conventional medicine treatment plans has been widely espoused. TCM is a treasure of ancient Chinese science. It may be one of the most resultful strategies to improve the efficacy of prevention and treatment of lung cancer recurrence and metastasis. Although studies have shown that patients treated with TCM have a higher risk of death [21], several studies have recently verified that adjuvant CHM treatments are beneficial to cancer patients' disease control [22–26]. The above studies showed that adjuvant CHM treatments not only has a positive effect on the survival time of patients, but also reduces the risk of recurrence and metastasis. However, there might be no significant difference in survival for elderly patients with advanced EGFR wild-type NSCLC who accepted CHM treatment. CHM has shown numerous benefits for the treatment of lung cancer including preventing recurrence and metastasis [27], reducing toxicity when taken in combination with chemotherapy as well as alleviating leucopenia [12, 22, 26], enhancing therapeutic effect in non-small cell lung cancer patients harboring EGFR mutations and improving quality of life [26, 28]. In addition, CHM could contribute to the recovery of immune function and improve TCM syndromes after operation [29]. To the best of our knowledge, CHM treatment should be given according to the patients' physical status, cancer category, and syndrome differentiation. In China, it has been demonstrated that CHM treatment could prolong PFS, improve immune functions, enhance chemotherapy toxicity, and improve quality of life [15, 16, 23], but its long-term efficacy remains largely unexplored until now.

This is the first study comparing the efficacy of long-term use of *Fuzheng Quxie Formula* with conventional medicine for patients with lung cancer in China. The study, starting January 1, 2005, was undertaken to assess PFS and OS in patients with lung cancer who were undergoing integrative treatments and conventional medicine alone. The samples were selected according to the uniform inclusion criteria, exclusion criteria, and strict follow-up to strengthen our evidence of research results. In this retrospective cohort study, there were significant survival benefits for patients receiving combined CHM and conventional medicine treatment compared with conventional medicine alone, especially after PSM. The above findings were supported by PSM in order to balance control and baseline confounding factors. In the present cohort, we divided the patients into the control group and the CHM group. Patients who received CHM treatment for more than six months experienced a preferable mPFS of 54.8 months, compared with 26.6 months in the control group. The main finding of our study, that CHM treatment could prolong PFS of lung cancer patients, is consistent with conclusions from the previous study [15, 30, 31]. It is also worth mentioning that our study was conducted in a larger sample and extended the follow-up time compared with the previous study [15]. Further study on the Cox multivariate regression also showed that the significant association between CHM application and improved PFS was independent of other factors of patient outcome, including gender, age, tumor stage, and surgery. It is also worth mentioning that there were no significant advantages in the survival curves of both stage III and stage IV lung cancer even after PSM. We speculated that the reason may be psychological factors. Research [21] found that approximately two-thirds of patients across the United States believe that CHM treatment will prolong life and one-third expect it to cure their diseases. Consequently, CHM treatment may result in inferior survival as a result of delays to receiving proven conventional treatments and refusal of other recommended treatments. We also speculate that this phenomenon is more common in China. On the other hand, patients with advanced lung cancer may interrupt conventional treatment by themselves during the treatment process. However, by further comparing the shape of survival plots, we have reached other conclusions. First of all, Kaplan–Meier curve of PFS for patients in different stages showed the CHM use could prolong PFS in the short term. Additionally, in spite of the fact that there was no statistical significance in log rank test of mOS after propensity score matching in stages II, the Kaplan–Meier curve of stage II OS still displayed a tiny advantage of CHM treatment.

The correlation between CHM exposure and PFS was further verified in the subgroup analysis. Our results indicated that the population may benefit most from CHM treatment. The HRs from patients subgroups were less than the total HRs, demonstrating that patients with specific characteristics may benefit more from CHM treatment. There remain several differences in how CHM and conventional western medicine diagnose and treat diseases. The foundation of TCM diagnosis takes the disease, syndrome, and symptoms into account, all of which could further form a treatment principle [32]. Therefore, syndrome differentiation is the core of TCM practice. As <Su Wen> goes, where evil-qi is gathered, the zheng-qi is certainly weak. We could also say that *zheng-qi* stands for vital energy for disease-resistant and upsets the equilibrium between yin and yang. The deficiency of *qi* could cause stasis of the blood, phlegm, and toxins, as well as the blockage of the meridians and viscera [33]. Based on the deficiency of *zheng-qi*, high viscosity of blood and formation of cancer plug are the important conditions for the recurrence and metastasis. Thus, to intervene in recurrence and metastasis, Professor Yan Li proposed the Fuzheng Quxie therapeutic principle for lung cancer, which attaches more emphasis to the Fuzheng principle than the Quxie principle. Among the 13 herbs in the *Fuzheng Quxie Formula*, there are 7 herbs for Fuzheng and 3 herbs for Quxie. Additionally, the Fuzheng principle includes invigorating qi, nourishing yin, enriching blood, and tonifying yang. In the prescription, Raw Astragalus (*Shenghuangqi*), Atractylodes Macrocephala (*Shengbaizhu*), and Rhizoma Dioscoreae (*Huaishanyao*) are used to strengthen qi and Fructus Lycii (*Gouqizi*), Radix Glehniae (*Beishashen*), Radix Ophiopogonis (*Maidong*), and Radix Glehniae (*Beishashen*) are applied for nourishing yin, while Semen Coicis (*Yiyiren*), Salvia Chinensis (*Shijianchuan*), Selaginella Doederleinii (*Shishangbai*), Oldenlandia Diffusa (*Sheshecao*), Poria Cocos (*Baifuling*), and Pericarpium Citri Reticulatae (*Chenpi*) were served for dispersing blood stasis and resolving phlegm. Throughout the whole prescription, the *Fuzheng Quxie Formula* plays an important role in the prevention and treatment of recurrence and metastasis in lung cancer. Our previous experimental research [34] found that *Fuzheng Quxie Formula* inhibited the growth and epithelial-mesenchymal transition (EMT) process of subcutaneous xenografts in Lewis lung cancer mice model and inhibited the phenotype and function of *M*2 macrophages. Besides, (-)-Guaiol, which is an effective constituent of the *Fuzheng Quxie Formula*, inhibited the EMT process of lung cancer by targeting *M*2 macrophages, and IL-10/STAT3 pathway is involved in the regulation of signaling pathway.

Nevertheless, there were also some limitations of our study. Firstly, the design was not a randomized controlled trial, which led to potential bias due to unrecognized confounding factors such as education, background, household income, and occupation. Secondly, due to deficiencies associated with a cohort study, a selection bias existed in the allocation of patients to the CHM and control groups [35]. Thirdly, our study was only conducted in Shanghai Pulmonary Hospital and Shanghai Municipal Hospital of Traditional Chinese Medicine because of the limited access to data. Fourthly, the small selective sample in control group limits the collection of OS data and the generalizability of the results to a broader population. Therefore, multicenter, double-blind, randomized, placebo-controlled trials need to be carried out to further verify the effects of CHM. In future retrospective clinical studies, the quality of follow-up should be improved. That is to say, we should ask whether the patient has stopped western medicine during treatment. If patients stop using conventional treatments for too long, we need to exclude such patients.

## 5. Conclusion

Long-term CHM treatment with the *Fuzheng Quxie Formula,* which can be flexibly applied in the course of lung cancer treatment, not only has a positive influence on the progression-free survival time of lung cancer patients, but also reduces the risk of recurrence and metastasis of lung cancer.

## Figures and Tables

**Table 1:** Clinical assessment of participants before and after PSM.

Item	Before PSM		*P* value	After PSM		*P* value
	CHM group (*n* = 761)	Control group (*n* = 373)		CHM group (*n* = 208)	Control group (*n* = 208)	
Gender						
Male	389 (51.1)	246 (66.7)	＜0.001^*∗*^	119 (57.2)	115 (55.3)	0.693
Female	372 (48.9)	128 (34.3)		89 (42.8)	93 (44.7)	
Age						
≤60	315 (41.4)	186 (49.9)	0.007^*∗*^	91 (43.8)	93 (44.7)	0.843
＞60	446 (58.6)	187 (50.1)		117 (56.2)	115 (55.3)	
Pathology						
Unspecified	43 (5.7)	1 (0.2)	＜0.001^*∗*^	1 (0.5)	1 (0.5)	0.927
Adenocarcinoma	546 (71.7)	235 (63.0)		151 (72.6)	143 (68.8)	
Squamous cell cancer	87 (11.4)	90 (24.1)		33 (15.9)	39 (18.8)	
Small cell lung cancer	29 (3.8)	31 (8.3)		12 (5.8)	12 (5.8)	
Others	56 (7.4)	16 (4.3)		11 (5.3)	13 (6.3)	
Stage						
I	295 (38.8)	193 (51.7)	＜0.001^*∗*^	99 (47.6)	93 (44.7)	0.842
II	39 (5.1)	35 (9.4)		18 (8.7)	22 (10.6)	
IIIA	47 (6.2)	56 (15.0)		23 (11.1)	29 (13.9)	
IIIB	41 (5.4)	51 (13.7)		28 (13.5)	27 (13.0)	
IV	339 (44.5)	37 (9.9)		40 (19.2)	37 (17.8)	
Surgery						
Yes	367 (48.2)	251 (67.3)	＜0.001^*∗*^	134 (64.4)	132 (63.5)	0.838
No	394 (51.8)	122 (32.7)		74 (35.6)	76 (36.5)	
Chemotherapy						
Yes	337 (44.3)	293 (78.6)	＜0.001^*∗*^	142 (68.3)	128 (60.1)	0.150
No	424 (55.7)	80 (21.4)		66 (31.7)	80 (38.5)	
Radiotherapy						
Yes	115 (15.1)	70 (18.8)	0.118	37 (17.8)	33 (15.9)	0.600
No	646 (84.9)	303 (81.2)		171 (82.2)	175 (84.1)	
Interventional therapy						
Yes	9 (1.2)	5 (1.3)	0.821	5 (2.4)	4 (1.9)	0.736
No	752 (98.8)	368 (98.7)		203 (97.6)	204 (98.1)	
Targeted therapy						
Yes	124 (16.3)	34 (9.1)	0.001^*∗*^	27 (13.0)	24 (11.5)	0.654
No	637 (83.7)	339 (90.9)		181 (87.0)	184 (88.5)	
Family history						
Yes	207 (27.2)	11 (2.9)	＜0.001^*∗*^	10 (4.8)	11 (5.3)	0.823
No	554 (72.8)	362 (97.1)		198 (95.2)	197 (94.7)	
Smoking						
Yes	251 (33.0)	99 (26.5)	0.027^*∗*^	63 (30.3)	71 (34.1)	0.401
No	510 (67.0)	274 (73.5)		145 (69.7)	137 (65.9)	
Drinking						
Yes	77 (10.1)	23 (6.2)	0.027^*∗*^	18 (8.7)	21 (10.1)	0.614
No	684 (89.9)	350 (93.8)		190 (91.3)	187 (89.9)	

PSM: propensity score matching; ^*∗*^*p* < 0.05, a significantly statistical difference in subgroup.

**Table 2:** Comparison of progression-free survival of two groups after PSM [case (%)].

Group	Cumulative recurrence/survival rate (%)									
	1-year	2-year	3-year	4-year	5-year	6-year	7-year	8-year	9-year	10-year
Control	61/70.56	105/49.18	121/41.01	129/36.54	139/30.72	146/25.58	150/21.23	152/18.65	152/18.65	152/18.65
CHM	27/86.88	54/73.17	72/63.15	79/58.63	86/53.06	91/48.12	93/45.32	95/41.62	95/41.62	96/39.02
*χ* ^2^	16.661	26.479	23.207	24.039	27.191	29.663	32.151	32.379	32.379	31.312
*P* value	<0.001^*∗*^	<0.001^*∗*^	<0.001^*∗*^	<0.001^*∗*^	<0.001^*∗*^	<0.001^*∗*^	<0.001^*∗*^	<0.001^*∗*^	<0.001^*∗*^	<0.001^*∗*^

^*∗*^*p* < 0.05, a significantly statistical difference between two groups.

**Table 3:** Cox regression results of lung cancer patients.

Factors	Univariate analysis					Multivariate analysis				
	*B*	Wald	*p*	HR	95%CI	*B*	Wald	*p*	HR	95%CI
CHM	−0.327	15.965	＜0.001	0.721	0.614–0.846	−0.774	53.138	＜0.001	0.461	0.375–0.568
Gender	0.852	98.220	＜0.001	2.343	1.980–2.773	0.347	11.359	0.001	1.414	1.156–1.730
Age	0.032	49.518	＜0.001	1.032	1.023–1.042	0.023	25.092	＜0.001	1.024	1.014–1.033
Pathology										
Adenocarcinoma	0.036	0.043	0.836	1.036	0.739–1.453					
Squamous cell cancer	−0.529	11.382	0.001	0.589	0.433–0.801					
Small cell lung cancer	0.185	0.675	0.411	1.204	0.774–1.872					
Others as control	NA	78.871	＜0.001	NA	NA					
Stage										
I as control	NA	269.917	＜0.001	NA	NA	NA	115.146	＜0.001	NA	NA
II	0.663	12.482	＜0.001	1.940	1.343–2.802	0.460	5.868	0.015	1.584	1.092–2.299
IIIA	1.295	80.139	＜0.001	3.650	2.749–4.846	0.882	32.380	＜0.001	2.417	1.783–3.275
IIIB	1.626	130.662	＜0.001	5.084	3.847–6.718	1.072	42.996	＜0.001	2.922	2.121–4.026
IV	1.620	242.732	＜0.001	5.054	4.122–6.197	1.437	112.673	＜0.001	4.208	3.227–5.486
Surgery	−0.997	148.926	＜0.001	0.369	0.314–0.433	−0.448	21.853	＜0.001	0.639	0.530–0.771
Chemotherapy	−0.343	61.861	＜0.001	0.710	0.651–0.773	−0.157	2.437	0.119	0.855	0.702–1.041
Radiotherapy	−0.365	61.164	＜0.001	0.694	0.634–0.761	0.059	0.332	0.564	1.061	0.867–1.298
Interventional therapy	0.458	2.062	0.151	1.581	0.846–2.955					
Targeted therapy	−0.339	47.864	＜0.001	0.712	0.647–0.784	−0.140	1.734	0.188	0.870	0.707–1.071
Family history	−0.339	9.413	0.002	0.712	0.574–0.885	0.028	0.059	0.807	1.029	0.820–1.291
Smoking	0.693	70.852	＜0.001	1.999	1.701–2.349	0.192	3.291	0.070	1.212	0.985–1.491
Drinking	0.600	23.410	＜0.001	1.822	1.429–2.323	0.191	1.879	0.170	1.210	0.921–1.589

## Data Availability

This two-center, retrospective cohort study collected clinical data from lung cancer patients who received treatments from the Shanghai Pulmonary Hospital and Shanghai Municipal Hospital of Traditional Chinese Medicine between January 1, 2005, and December 30, 2020. All patients were followed from the date of diagnosis to the date of death or up to December 30, 2020. The date of death was determined from a database maintained by the Shanghai Municipal Center for Disease Control and Prevention of Cancer Patient Registration System. Additionally, telephone and clinical in-person visits were performed every six months.

## Abbreviations

CHM: Chinese herbal medicine
PSM: Propensity score matching
OS: Overall survival
PFS: Progression-free survival
HR: Hazard ratio
EMT: Epithelial-mesenchymal transition.
